# Utility and Safety of Endosonography in the Diagnosis of Small Cell Lung Cancer: A Prospective Single-Center Observational Study

**DOI:** 10.3390/diagnostics16091294

**Published:** 2026-04-26

**Authors:** Carmine Salerni, Silvia Terraneo, Michele Bonanomi, Sara Mirijaj, Cristina Albrici, Giulia Carone, Letizia Gianoncelli, Mauro Moroni, Umberto Gianelli, Guido Marchi, Paolo Carlucci, Michele Mondoni

**Affiliations:** 1 Respiratory Unit, ASST Santi Paolo e Carlo, 20142 Milan, Italy; silvia.terraneo@asst-santipaolocarlo.it (S.T.);; 2 Oncology Unit, ASST Santi Paolo e Carlo, 20142 Milan, Italy; 3 Department of Health Sciences, Università Degli Studi di Milano, 20126 Milan, Italy; 4 Pathology Unit, ASST Santi Paolo e Carlo, 20142 Milan, Italy; 5 Pulmonology Unit, University Hospital of Pisa, 56100 Pisa, Italy; 6 Respiratory Unit, ASST Ovest Milanese, 20025 Legnano, Italy

**Keywords:** endoscopic ultrasound with bronchoscope fine-needle aspiration, EUS-B-FNA, endobronchial ultrasound-guided transbronchial needle aspiration, EBUS-TBNA, small cell neuroendocrine lung cancer

## Abstract

**Background**: Endosonography (i.e., endoscopic ultrasound with bronchoscope fine-needle aspiration, EUS-B-FNA and endobronchial ultrasound-guided transbronchial needle aspiration, EBUS-TBNA) is a widely used technique in the diagnosis and staging of non-small cell lung cancer. Limited data are available in diagnosing small cell lung cancer (SCLC), and no studies have specifically investigated the diagnostic accuracy of EUS-B-FNA in these patients. The study aims at evaluating the sensitivity and safety of endosonography in the diagnosis of SCLC. **Methods**: A prospective, single-center, observational study was conducted in Italy. All patients diagnosed with SCLC who underwent EUS-B-FNA and/or EBUS-TBNA were enrolled. The sensitivity of EUS-B-FNA and EBUS-TBNA were assessed using pathological confirmation as the gold standard. **Results**: A total of 72 patients (38 (53%) males) with confirmed SCLC were included in the study. EUS-B-FNA was performed in 31 (43%) patients and EBUS-TBNA in 44 (61.1%) patients; both procedures were performed in three (4.2%). The overall sensitivity of endosonography was 97.2%. The sensitivity of EUS-B-FNA and EBUS-TBNA was 96.8% and 90.9%, respectively. No differences were observed in the sensitivity of both techniques when sampling lymph nodes vs. pulmonary parenchymal lesions (*p* = 0.99). The overall complication rate was 5.6%. No major complications were reported. **Conclusions**: Endosonography is a highly accurate and safe technique in diagnosing SCLC. EUS-B-FNA alone demonstrates excellent sensitivity, supporting its extensive role as a valuable diagnostic tool. The combined use of both techniques may further optimize diagnostic yield in the diagnosis of SCLC.

## 1. Introduction

Small cell lung cancer (SCLC) is a high-grade neuroendocrine carcinoma that represents about 15% of lung cancers, with a prevalence of 1–5 per 10,000 individuals in the European population [1]. It is characterized by an exceptionally short doubling time, often in the range of a few weeks, and a high propensity for early dissemination leading to frequent presentation at an advanced stage [2]. Despite the introduction of immunotherapy in association with chemotherapy in first line setting for extensive-stage or as adjuvant treatment after chemoradiotherapy for limited disease, rapid treatment resistance still leads to poor long-term outcomes, with a 5-year survival rate of less than 10% [3,4,5,6]. Recent molecular studies have identified four SCLC subtypes defined by differential expression of transcription factors ASCL1, NEUROD1, and POU2F3, or low expression of all three accompanied by an inflamed gene signature (SCLC-A, -N, -P, and -I, respectively). Analysis of IMpower133 trial samples demonstrated that the SCLC-I subtype may experience the greatest benefit from the addition of immunotherapy to chemotherapy [7]. These observations underscore the importance of obtaining adequate tissue at diagnosis, not only for histological confirmation but also for potential future molecular characterization. Given its typical radiological presentation—a central hilar mass with bulky mediastinal lymphadenopathy in close contact with the airways and/or the esophagus—SCLC appears particularly suited for diagnosis by endosonography [8]. Endobronchial ultrasound-guided transbronchial needle aspiration (EBUS-TBNA) and endoscopic ultrasound with bronchoscope transesophageal fine-needle aspiration (EUS-B-FNA) are well-established techniques for sampling malignant and benign mediastinal/hilar lymph nodes and central parenchymal lesions [9,10,11]. In non-small cell lung cancer (NSCLC), combining the transbronchial and transesophageal approaches improves diagnostic accuracy of mediastinal staging, and the transesophageal route is generally better tolerated, with shorter procedure times and fewer adverse events, making it particularly suitable for frail patients [9,12,13,14,15]. Despite these advantages, only a limited number of studies have specifically assessed the accuracy of endosonographic techniques in the initial diagnosis of SCLC [16,17,18,19]. Moreover, EUS-B-FNA has been poorly studied in the diagnosis of these patients [20]. Therefore, the diagnostic performance, tissue adequacy, and safety profile of endosonography in SCLC remain incompletely defined. Addressing this knowledge gap is clinically relevant because rapid, minimally invasive, and accurate diagnostic strategies may facilitate earlier treatment initiation and may provide sufficient material for potential future molecular characterization. To our knowledge, this is the first prospective study specifically evaluating the use of EBUS-TBNA and EUS-B-FNA for the diagnosis of SCLC. The primary aim of this study was to evaluate the diagnostic accuracy of endosonography (i.e., EBUS-TBNA and EUS-B-FNA) in the diagnosis of SCLC. The main predictors of success and the safety of the techniques have also been assessed.

## 2. Materials and Methods

### 2.1. Study Design

A prospective, observational, single-center study was carried out at ASST Santi Paolo e Carlo, Milan, Italy. The study protocol was approved by the institutional ethics committee (Comitato Etico Milano Area 1; approval number 7206/2019). Written informed consent was obtained from all patients.

### 2.2. Patients and Interventions

All the patients with a confirmed pathological diagnosis of SCLC who underwent EUS-B-FNA and/or EBUS-TBNA between 1 March 2019 and 31 May 2025 were consecutively included in the analysis. In cases of negative or inadequate cytopathological findings from endosonography, a definitive histopathological diagnosis was obtained via alternative diagnostic procedures, including conventional bronchoscopy, transthoracic needle aspiration, video-assisted thoracoscopic surgery (VATS), or thoracotomy. All patients underwent a standard diagnostic work-up, including medical history, physical examination, laboratory testing (complete blood count, serum creatinine, blood urea nitrogen, prothrombin time, and activated partial thromboplastin time), contrast-enhanced chest computed tomography (CT), and positron emission tomography (PET)/CT. Patients were reassessed clinically 24 h post-procedure to detect delayed complications. All procedures were performed by experienced interventional pulmonologists with more than 15 years of expertise in endosonography, under local anesthesia with conscious (midazolam/fentanyl) or deep sedation (propofol/remifentanil). Due to the lack of standardized international guidelines, procedural decisions, such as needle gauge, number of passes, aspiration technique, use of, and need for both EUS-B-FNA and EBUS-TBNA, were left to the bronchoscopist’s discretion based on clinical context. Rapid on-site evaluation (ROSE), performed by a pathologist, was used in all the procedures.

Data collected included: patient demographics (age, sex, and nationality), clinical parameters (BMI, smoking history, and presenting symptoms), disease characteristics (stage, involvement of other organs/systems), procedural details (number, location, and size of sampled lymph nodes; needle gauge; use of ROSE; sample adequacy; and reason for choosing EUS-B-FNA), adverse events occurring during or within 24 h post-procedure, final histopathological diagnosis, and reference standard used in nondiagnostic cases.

### 2.3. Definition of Sample Adequacy and Accuracy of the Test

A fine-needle aspiration sample was considered adequate if the material obtained was sufficient for a definite cytopathological diagnosis. On the contrary, according to the 5th edition of the World Health Organization, a specimen is categorized as “Insufficient/Inadequate/Non-diagnostic” when the cytopathology material does not contain any diagnostic material that could explain the imaging findings or clinical symptoms [21]. Moreover, ROSE was considered adequate if the material was sufficient to confirm that the specimen was obtained from a lymph node/pulmonary lesion.

### 2.4. Outcome Measures

The primary outcome was the sensitivity, defined as the proportion of patients with a final diagnosis of SCLC correctly identified by endosonography, using histopathological or clinical confirmation as the reference standard. Since only patients with a final diagnosis of small cell lung cancer were included, false-positive cases could not be evaluated and specificity and negative predictive value could not be calculated. Secondary outcomes included accuracy of endosonography related to lymph node/parenchymal lesions sampling, lymph node station and lesions’ size and evaluation of the safety of the technique.

### 2.5. Statistical Analysis

Continuous variables were summarized using mean ± standard deviation (SD) or median and interquartile range (IQR), as appropriate. Categorical variables were presented as absolute and relative frequencies and were compared using the Chi-square test or the Fisher exact test. A *p* value < 0.05 was considered statistically significant. Sensitivity and accuracy were calculated using the histopathological or clinical reference standard. Statistical analyses were performed with SPSS version 29.02.

## 3. Results

**Figure 1:**
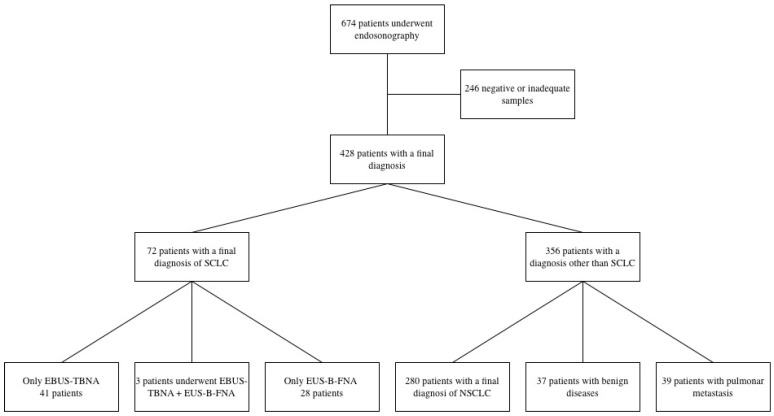
Study flow chart.

A total of 674 patients underwent thoracic endosonography at the Respiratory Unit of ASST Santi Paolo e Carlo between 1 March 2019 and 31 May 2025 (Figure 1). A total of 72 adult patients (38 (53%) males, mean age ± standard deviation (SD) of 69.5 (±9.8 years)), who had a final diagnosis of SCLC were enrolled (Table 1). In 31 (43%) patients, EUS-B-FNA was performed, while 44 (61%) patients underwent EBUS-TBNA. A combined approach using both EUS-B-FNA and EBUS-TBNA was employed in 3 (4.2%) of these cases. A 22-gauge needle was used for all patients. The mean number of needle passes per procedure was 4.2 ± 1.7. EUS-B-FNA was performed when EBUS-TBNA was not feasible due to anesthesiological reasons (21 patients, 70%) or persistent cough (six patients, 20%), when the lesion was not accessible with EBUS-TBNA (two patients, 7%), or when previous EBUS-TBNA sampling was inadequate (one patient, 3%) (Table 2).

**Figure 2:**
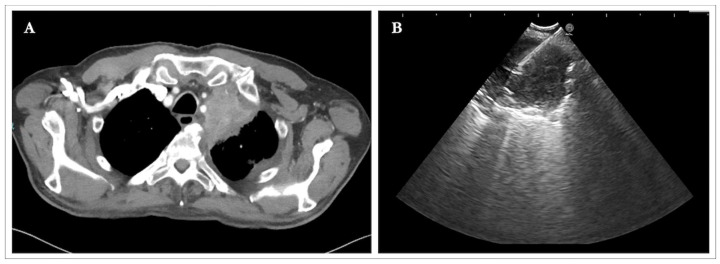
Radiological–endosonographic correlation of a parenchymal lesion sampled by EUS-B-FNA. (**A**) Chest CT scan showing a lung lesion in the left upper lobe. (**B**) Endosonographic image of a EUS-B-FNA of the malignant lesion.

Representative radiological and endosonographic images of a parenchymal lesion sampling via the transesophageal approach (EUS-B-FNA) are shown in Figure 2.

On-site cytopathological evaluation (ROSE) was performed in all procedures, with adequate sampling achieved in 100% of the cases.

The overall sensitivity of endosonography in the diagnosis of SCLC was 97.2% (95% CI 90.3–99.7). Sensitivity of EUS-B-FNA was 96.8% (95% CI 83.3–99.9), while EBUS-TBNA had a sensitivity of 90.9% (95% CI 78.3–97.4) (Table 3). In three patients, in whom both procedures were performed during the same session, a combined sensitivity of 100% was recorded. Endosonography (EUS-B-FNA and/or EBUS-TBNA) was not diagnosed in two (2.7%) patients. In these, definitive diagnosis of SCLC was established via bronchoscopy with endobronchial biopsy (EBB) in one patient and CT-guided transthoracic biopsy in another patient.

Endosonography (EUS-B-FNA and EBUS-TBNA) was diagnostic in all patients with lymph nodes larger than 2 cm whereas in 2 out of 3 (66.7%) with lymph nodes <1 cm (*p* = 0.007). All cases in which the parenchymal lesion was sampled (all larger than 3 cm) resulted diagnostic (Table 4).

No differences were observed in the sensitivity of both techniques when sampling lymph nodes vs. pulmonary parenchymal lesions (*p* = 0.99) (Tables S1 and S2 Supplemental Digital Content).

The overall complication rate was 5.6%. Among patients who underwent exclusive EUS-B-FNA, two (6.5%) developed post-procedural, transient chest pain and fever within 24 h, both of which resolved spontaneously without requiring medical intervention. In the EBUS-TBNA group, two (4.5%) patients experienced complications as follows: one had a hypertensive peak during the procedure, while the other reported chest pain within 24 h post-procedure. Additionally, one (2.3%) patient who underwent EBUS-TBNA required hospitalization due to acute exacerbation of chronic obstructive pulmonary disease (COPD) ten days after the procedure. Notably, three patients who underwent both procedures in the same session did not experience any adverse event.

## 4. Discussion

This is one of the largest studies which aimed at assessing the utility of endosonography in the diagnosis of patients with SCLC. We first demonstrated, on a large number of patients, the high accuracy of EUS-B-FNA in the diagnostic work-up of these patients. Our study confirms that endosonography is an accurate and safe technique for diagnosing SCLC, with performance characteristics that align with the existing literature [9,10,11].

A limited number of studies have specifically assessed the accuracy of endosonographic techniques, particularly EBUS-TBNA, in the initial diagnosis of SCLC. They showed a high sensitivity (ranging from 96.4% to 98.5%) in the diagnosis and mediastinal staging [16,17,18,19].

Kang et al., in a retrospective observational study involving 161 patients with SCLC, reported an overall sensitivity of 97.4% for endosonography [18]. He first described five patients with SCLC who were diagnosed by EUS-B-FNA, showing a sensitivity of 100%. The low number of patients who underwent this technique limit the possibility of drawing conclusions about its standalone diagnostic performance.

The existing literature acknowledges the complementary role of EBUS-TBNA and EUS-B-FNA in mediastinal staging; however, a very limited number of studies have systematically evaluated their combined diagnostic yield in SCLC. In our study, 31 patients underwent EUS-B-FNA, with a diagnostic yield of 96.8%, which was superior to that of EBUS-TBNA (90.9%). Notably, EUS-B-FNA enabled the diagnosis of SCLC in 30 (41.7%) patients for whom EBUS-TBNA could not be performed for clinical and/or anatomical reasons or retrieved an inadequate sample. Compared with previous studies including heterogeneous lung cancer populations or limited numbers of SCLC cases, our study provides prospective real-world evidence on the diagnostic performance of EBUS-TBNA and EUS-B-FNA specifically in patients with SCLC. These findings highlight that the combination of both techniques may significantly enhance the diagnostic accuracy of bronchoscopy for the diagnosis of SCLC, thus reinforcing the value of a multimodal endosonographic approach. Additionally, we provide real-world evidence supporting the use of EUS-B-FNA in specific clinical scenarios, such as patients with poor tolerance or contraindications to EBUS-TBNA (e.g., in patients with persistent cough or respiratory failure), or lesions inaccessible via the transbronchial route.

To date, no studies in the scientific literature have assessed the predictive variables for the success of endosonography (i.e., EBUS-TBNA and/or EUS-B-FNA) in diagnosing SCLC. Our study demonstrated that the high diagnostic accuracy of endosonography is independent of anatomical, endoscopic, and pathological variables.

The high sensitivity and specificity of endoscopic ultrasound needle aspiration techniques in diagnosing SCLC can be attributed to the central location of the tumor in most cases, the average large size of the target lesions, and the characteristic cytological features, i.e., the presence of small cells with scant cytoplasm, and the alteration in cellular attachment as reflected in decreased cellular cohesion which may favor needle aspiration sampling [22]. Ren et al. reported a high accordance rate of cytological subtyping for small-cell carcinoma on EBUS-TBNA specimens (98.1%), supporting the reliability of cytological diagnosis in this tumor type [23]. In contrast, cytological typing of NSCLC may be more challenging in a subset of cases and may require ancillary techniques when limited material is available [24]. Furthermore, early-stage lung adenocarcinoma frequently presents as peripheral subsolid nodules, which may be less accessible to endosonographic sampling and may partly contribute to the lower diagnostic yield reported in NSCLC compared with SCLC [25].

In our study, rapid on-site evaluation (ROSE) was used in all cases. A complete concordance between ROSE findings and the final pathological diagnosis of SCLC was observed, with no false positives. These data confirm the utility of immediate cytopathological assessment which provides valuable information on quality and quantity of malignant cells, crucial to guide the sampling strategy.

We described a low rate of adverse events, all resolved without the need for medical intervention, confirming the safety of the technique [15]. By comparison, CT-guided transthoracic needle biopsy, which represents the main alternative percutaneous diagnostic approach for lung lesions, has been reported to be associated with pneumothorax rates ranging from 18% to 28% and pulmonary hemorrhage in approximately 27% to 33% of cases. Importantly, hilar and mediastinal lesions—which represent the typical presentation of SCLC—can be challenging to access percutaneously due to the proximity to major vascular structures, and robust data on diagnostic yield and complication rates for CT-guided biopsy of central lesions are lacking [26].

Serious complications such as esophageal perforation, mediastinitis, and fistula formation have been described with conventional endoscopic ultrasound-guided fine needle aspiration (EUS-FNA), although these events appear to be exceedingly rare [27,28]. In our cohort, no cases of esophageal perforation, mediastinitis, or fistula formation were observed. This observation is consistent with the prospective studies evaluating EUS-B-FNA, including the randomized study by Oki et al., which reported no major complications related to esophageal injury [15].

Our study has several limitations. First, this was a prospective single-center study, which may limit the external validity and generalizability of the findings. In the absence of international guidelines, a standardized protocol for the procedure was not implemented, and clinical and procedural decisions depended on operator experience and clinical judgment. The choice of the endoscopic technique was based on operator preference; consequently, the two groups may not represent fully comparable populations, and comparisons between them should be interpreted with caution due to the potential for selection bias. Additionally, some procedural details, such as the aspiration technique, were not systematically recorded.

Furthermore, the relatively small sample size of both the EUS-B-FNA and EBUS-TBNA groups—and particularly the very limited number of patients who underwent the combined procedure—may have reduced the statistical power and introduced potential procedural bias. Similarly, the small number of patients in the non-diagnostic group may limit the robustness of the statistical comparisons, including the diagnostic performance based on lymph node size. Finally, since only patients with a final diagnosis of small cell lung cancer were included, false-positive cases could not be evaluated, and specificity and negative predictive value could not be calculated. Therefore, our results should be interpreted with caution and confirmed in larger, prospective, multicenter studies.

In conclusion, our study confirms the safety and accuracy of endosonography in the pathological diagnosis of SCLC. EBUS-TBNA and EUS-B-FNA are complementary endoscopic techniques, and the ability to perform both, based on information provided by ROSE, may contribute to optimizing the diagnostic yield of bronchoscopy in selected clinical scenarios. However, given the limited number of patients who underwent the combined approach in our cohort, no definitive conclusions regarding its potential benefit can be drawn.

## 5. Conclusions

Given the high diagnostic yield and favorable safety profile observed in our study, EUS-B-FNA appears to be a valuable tool in the diagnostic algorithm of SCLC, particularly in cases where conventional bronchoscopy is not feasible or EBUS is poorly tolerated.

However, multicentre prospective studies are needed to confirm these results and define a standardized protocol for the optimal combination of endosonographic techniques in the diagnosis of SCLC.

## Figures and Tables

**Table 1:** Demographic and clinical characteristics of enrolled patients.

Demographic and Clinical Variables	
Age, years (mean ± SD)	70 (10)
Sex (male), *n* (%)	38 (53)
Ethnicity, *n* (%)	
Caucasian	68 (94)
African	4 (6)
Smoking history—never smoker/smoker/former smoker, *n*%	2 (3)/28 (39)/42 (58)
Pack years (mean ± SD)	42 (10)
Chronic pulmonary diseases	
Obstructive lung diseases, *n* (%)	23 (32)
IPF, *n* (%)	2 (3)
Chronic heart disease, *n* (%)	14 (19)
Chronic liver disease, *n* (%)	2 (3)
Chronic kidney failure, *n* (%)	1 (1)
Diabetes, *n* (%)	17 (24)
Lesion site, *n* (%)	
Upper right lobe	24 (33)
Medium lobe	14 (20)
Upper left lobe	17 (24)
Lower left lobe	10 (14)
Lower right lobe	19 (26)
Parenchymal lesion, size at CT scan (largest diameter), n (%)	
<3 cm	10 (14)
3–7 cm	30 (42)
>7 cm	32 (44)
Lymphadenopathy, size at CT scan (shortest diameter), n (%)	
<1 cm	3 (4)
1–2 cm	18 (25)
>2 cm	51 (71)
Time from procedure to cytopathological diagnosis, days (mean ± SD)	5 (3)
Final stage of SCLC (VALSG), n (%)	
Limited disease	23 (32)
Extensive disease	49 (68)

Data are expressed as number and numbers with percentages; SD: standard deviation, TNM classification according to 8th Edition of the TNM Classification for Lung Cancer; COPD: chronic obstructive pulmonary disease; IPF: idiopathic pulmonary fibrosis. CT, computed tomography; VALSG, Veterans Administration Lung Study Group. Parenchymal lesion and lymph node size were measured on chest CT.

**Table 2:** Main procedural characteristics.

Indication to Perform EUS-B-FNA, n (%)	
Lesion not accessible with EBUS-TBNA	2 (7)
Sampling not adequate at a previous EBUS-TBNA	1 (3)
EBUS-TBNA not performed due to anesthesiological reasons	21 (70)
Persistent cough	6 (20)
Number of lymph node stations sampled, n (%)	
One lymph node station	66 (94)
Two lymph node stations	4 (6)
Sampling site, n (%)	
Lung parenchymal lesions	13 (18)
Lymph nodes (station number)	
Subcarinal lymph nodes (7)	28 (39)
Right lower paratracheal lymph nodes (4R)	15 (21)
Right interlobar lymph nodes (11R)	2 (3)
Right hilar lymph nodes (12R)	1 (1)
Left lower paratracheal lymph nodes (4L)	3 (4)
Left hilar lymph nodes (10L)	3 (4)
Right hilar lymph nodes (10R)	4 (6)
Left upper paratracheal lymph nodes (2L)	1 (1)
Additional sampling site, n (%)	
Lung parenchymal lesion	1 (20)
Subcarinal lymph node (7)	1 (20)
Right lower paratracheal lymph node (4R)	2 (40)
Left lower paratracheal lymph node (4L)	1 (20)

Data are expressed as number and numbers and percentages. The target sampling procedure may be EBUS-TBNA or EUS-B-FNA. Some patients underwent sampling of more than one site during the same procedure. EUS-B-FNA: endoscopic ultrasound with bronchoscope-guided fine needle aspiration; EBUS-TBNA: endobronchial ultrasound-guided transbronchial needle aspiration.

**Table 3:** Diagnostic performance of endosonography in the diagnosis of small cell neuroendocrine lung.

Target	Technique	Sensitivity *N*, % (95%CI)
Overall	EUS-B-FNA or EBUS-TBNA	70/72, 97.2% (90.3–99.7)
	EUS-B-FNA	30/31, 96.7% (83.3–99.9)
	EBUS-TBNA	40/44, 90.9% (78.3–97.4)
	EUS-B-FNA and EBUS-TBNA	3/3, 100% (29.2–100)
Parenchymal lesion	EUS-B-FNA or EBUS-TBNA	13/13, 100% (75.3–100.0)
	EUS-B-FNA	10/10, 100% (69.2–100.0)
	EBUS-TBNA	3/3, 100% (29.2–100.0)
Lymph nodes	EUS-B-FNA or EBUS-TBNA	57/59, 96.6% (88.3–99.6)
	EUS-B-FNA	20/21, 95.2% (76.2–99.9)
	EBUS-TBNA	37/41, 90.2% (76.8–97.3)
	EUS-B-FNA and EBUS-TBNA	3/3, 100% (29.2–100.0)

Diagnostic performance was calculated according to the number of procedures performed. EUS-B-FNA: endoscopic ultrasound with bronchoscope-guided fine needle aspiration; EBUS-TBNA: endobronchial ultrasound-guided transbronchial needle aspiration, CI: confidence interval.

**Table 4:** Diagnostic performance of endosonography in the diagnosis of small cell neuroendocrine lung according to patient, lesion, and procedural characteristics.

	Diagnostic Endosonography	Non-Diagnostic Endosonography	*p*-Value
Number of patients, n (%)	70 (97)	2 (3)	
Sex (male), n (%)	36 (50)	2 (100)	0.275
Comorbidity, n (%)			
Diabetes	17 (25)	0 (0)	0.576
Chronic renal disease	1 (1)	0 (0)	0.972
Chronic heart disease	13 (19)	1 (50)	0.358
Chronic lung disease **	24 (34)	1 (50)	0.577
Lymph node stations, n (%)			
Subcarinal (Station 7)	27 (39)	1 (50)	0.995
Right lower paratracheal (Station 4R)	14 (20)	1 (50)	
Left lower paratracheal (Station 4L)	3 (4)	0 (0)	
Left hilar (Station 10L)	3 (4)	0 (0)	
Right hilar (Station 10R)	4 (6)	0 (0)	
Right interlobar (Station 11R)	2 (3)	0 (0)	
Left interlobar (Station 11L)	2 (3)	0 (0)	
Right lobar (Station 12R)	1 (1)	0 (0)	
Left upper paratracheal (Station 2L)	1 (1)	0 (0)	
Lung parenchymal lesion	13 (8)	0 (0)	
Sampled lymph node diameter, n (%)			
<1 cm	2 (4)	1 (50)	0.007
1–2 cm	15 (26)	1 (50)	
>2 cm	40 (70)	0 (0)	
Sampled parenchymal lesion diameter, n (%) 3–7 cm >7 cm			
	5 (39) 8 (61)	0 (0) 0 (0)	-
Final staging, n (%)			
Limited disease	23 (33)	0 (0)	0.999
Extensive disease	47 (67)	2 (100)	

Data are expressed as number and numbers and percentages. ** intended as patients with chronic obstructive pulmonary disease or idiopathic pulmonary fibrosis. Diagnostic performance was calculated according to the number of patients who underwent endosonography.

## Data Availability

The raw data supporting the conclusions of this article will be made available by the authors on request.

## Supplementary Materials

The following supporting information can be downloaded at https://www.mdpi.com/article/10.3390/diagnostics16091294/s1, Table S1: Demographic and clinical characteristics of enrolled patients according to EUS-B-FNA sensitivity; Table S2: Demographic and clinical characteristics of enrolled patients according to EBUS-TBNA sensitivity.

## Abbreviations

The following abbreviations are used in this manuscript:

SCLC	Small Cell Lung Cancer
EUS-B-FNA	Endoscopic Ultrasound with Bronchoscope-Guided Fine-Needle Aspiration
EBUS-TBNA	Endobronchial Ultrasound-Guided Transbronchial Needle Aspiration
NSCLC	Non-Small Cell Lung Cancer
VATS	Video-Assisted Thoracoscopic Surgery
CT	Computed Tomography
PET	Positron Emission Tomography
ROSE	Rapid On-Site Evaluation
BMI	Body Mass Index
IPF	Idiopathic Pulmonary Fibrosis
COPD	Chronic Obstructive Pulmonary Disease
VALSG	Veterans Administration Lung Study Group
TNM	Tumor Node Metastasis (Classification System)
SD	Standard Deviation
IQR	Interquartile Range
CI	Confidence Interval
SPSS	Statistical Package for the Social Sciences
