# Supplementary material for: Utility and Safety of Endosonography in the Diagnosis of Small Cell Lung Cancer: A Prospective Single-Center Observational Study
Source: Diagnostics (Basel). 2026 Apr 26;16(9):1294. doi: 10.3390/diagnostics16091294 (PMC13163117; doi:10.3390/diagnostics16091294)
Supplement: Supplementary file 1 [file diagnostics-16-01294-s001.zip › diagnostics-4191482-supplementary.pdf]

Table S1. Demographic and clinical characteristics of enrolled patients according to EUS-B-FNA sensitivity

	Diagnostic EUS-B-FNA	Non-diagnostic EUS-B-FNA	*P*- value
Number of procedures, n (%)	30 (96.8)	1 (3.2)	
Sex (male), n (%)	10 (33)	1 (100)	0.333
Comorbidity, n (%)			
Diabetes	8 (28)	0 (0)	>0.99
Chronic renal disease	1 (3)	0 (0)	>0.99
Chronic heart disease	8 (28)	0 (0)	>0.99
COPD	10 (33)	0 (0)	>0.99
Sampled site, n (%)			
Subcarinal (Station 7)	17 (57)	0 (0)	<0.001
Left lower paratracheal (Station 4L)	3 (10)	0 (0)	
Left hilar (Station 10L)	0 (0)	1 (100)	
Lung parenchymal lesion	10 (33)	0 (0)	
Sampled lymph node diameter, n (%)			
<1 cm	1 (3)	0 (0)	0.883
1-2 cm	5 (16)	0 (0)	
>2 cm	24 (80)	1 (100)	
Sampled parenchymal lesion diameter, n (%)			
3-7cm	5 (50)	0 (0)	-
>7 cm	5 (50)	0 (0)	
Final staging, n (%)			
Limited disease	7 (23)	0 (0)	>0.99
Extensive disease	23 (76)	1 (100)	

Data are expressed as number and numbers and percentages; supplementary tables report procedure-based data; three patients underwent both procedures and therefore appear in both tables; COPD: chronic obstructive pulmonary disease.

Table S2. Demographic and clinical characteristics of enrolled patients according to EBUS-TBNA sensitivity

	Diagnostic EBUS-TBNA	Non-diagnostic EBUS-TBNA	P-value
Number of procedures, n, (%)	40 (90.9)	4 (9.1)	
Sex (male), n (%)	26 (65)	3 (75)	0.579
Comorbidity, n (%)			
Chronic heart disease	5 (12)	1 (25)	0.456
COPD	12 (30)	1 (25)	0.999
Sampled site			
Left upper paratracheal (Station 2L)	1 (2.5)	0 (0)	
Subcarinal (Station 7)	9 (23)	3 (75)	
Right lower paratracheal (Station 4R)	14 (35)	1 (25)	
Left hilar (Station 10 L)	3 (7.5)	0 (0)	
Right hilar (Station 10 R)	4 (10)	0 (0)	0.705
Left interlobar (Station 11L)	2 (5)	0 (0)	
Right interlobar (Station 11R)	2 (5)	0 (0)	
Right interlobar (Station 12R)	1 (2.5)	0 (0)	
Lung parenchymal lesion	4 (10)	0(0)	
Sampled lymph node (diameter)			
<1 cm	1 (3)	1 (25)	
1-2 cm	12 (30)	1 (25)	0.119
>2 cm	27 (67)	2 (50)	
Sampled parenchymal lesion (diameter)			
>7 cm	4 (100)	-	
Final staging			
Limited disease	16 (40)	1 (25)	0.496
Extensive disease	24 (60)	3 (75)	

Data are expressed as number and numbers and percentages; supplementary tables report procedure-based data; three patients underwent both procedures and therefore appear in both tables; COPD: chronic obstructive pulmonary disease.
